# Fatigue, induced via repetitive upper-limb motor tasks, influences trunk and shoulder kinematics during an upper limb reaching task in a virtual reality environment

**DOI:** 10.1371/journal.pone.0249403

**Published:** 2021-04-08

**Authors:** Frédérique Dupuis, Gisela Sole, Craig Wassinger, Mathieu Bielmann, Laurent J. Bouyer, Jean-Sébastien Roy

**Affiliations:** 1 Faculty of Medicine, Université Laval, Quebec City, Canada; 2 Centre for Interdisciplinary Research in Rehabilitation and Social Integration, Quebec City, Canada; 3 Centre for Health, Activity and Rehabilitation Research, School of Physiotherapy, University of Otago, Dunedin, New Zealand; 4 Department of Physical Therapy, East Tennessee State University, Johnson City, TN, United States of America; Toronto Rehabilitation Institute - UHN, CANADA

## Abstract

**Background:**

Efficient shoulder movement depends on the ability of central nervous system to integrate sensory information and to create an appropriate motor command. Various daily encountered factors can potentially compromise the execution of the command, such as fatigue. This study explored how fatigue influences shoulder movements during upper limb reaching.

**Methods:**

Forty healthy participants were randomly assigned to one of two groups: Control or Fatigue Group. All participants completed an upper limb reaching task at baseline and post-experimental, during which they reached four targets located at 90° of shoulder abduction, 90° external rotation at 90° abduction, 120° scaption, and 120° flexion in a virtual reality environment. Following the baseline phase, the Fatigue Group completed a shoulder fatigue protocol, while Controls took a 10-minute break. Thereafter, the reaching task was repeated. Upper limb kinematic (joint angles and excursions) and spatiotemporal (speed and accuracy) data were collected during the reaching task. Electromyographic activity of the anterior and middle deltoids were also collected to characterize fatigue. Two-way repeated-measures ANOVA were performed to determine the effects of Time, Group and of the interaction between these factors.

**Results:**

The Fatigue group showed decreased mean median power frequency and increased electromyographic amplitudes of the anterior deltoid (p < 0.05) following the fatigue protocol. Less glenohumeral elevation, increased trunk flexion and rotation and sternoclavicular elevation were also observed in the Fatigue group (Group x Time interaction, p < 0.05). The Control group improved their movement speed and accuracy in post-experimental phase, while the Fatigue group showed a decrease of movement speed and no accuracy improvement (Group x Time interaction, p < 0.05).

**Conclusion:**

In a fatigued state, changes in movement strategy were observed during the reaching task, including increased trunk and sternoclavicular movements and less glenohumeral movement. Performance was altered as shown by the lack of accuracy improvement over time and a decrease in movement speed in the Fatigue group.

## Introduction

The main role of the shoulder is to position the hand in space to perform daily life activities (*i*.*e*. reaching, eating, playing sports, carrying objects, etc.) [1, 2]. To enable such activities, a high level of mobility is required. The glenohumeral joint is the most mobile joint of the body due to its numerous degrees of freedom [2–4]. While its anatomical configuration provides this high level of mobility, it makes preserving glenohumeral stability a challenge, especially in elevated arm positions (e.g. more than 60° of elevation) [1, 2]. Passive (*i*.*e*. ligaments, articular surfaces and labrum) and active (*i*.*e*. muscles) structures contribute to the stability of the shoulder [5, 6]. The numerous sensory receptors (*e*.*g*. muscle spindles, Golgi tendon organs, Ruffini endings, Pacinian and Meissner corpuscles) contained in these structures continuously relay sensory information to the central nervous system (CNS) on movement being performed, limbs’ position and forces applied to periarticular structures [4, 7]. The CNS integrates this sensory information with a desired movement trajectory to create an appropriate motor command that will ensure efficient shoulder movement and stability while completing a given task [2, 4, 7, 8].

Altered shoulder movement has been proposed as an etiological factor for the development and maintenance of shoulder pain, such as rotator cuff related shoulder pain (RCRSP). Indeed, individuals with chronic RCRSP present with changes in their glenohumeral and sternoclavicular inter-joint coordination during upper limb movement [9–11]. Inter-joint coordination refers to the coordination between two or more joints when performing functional movements such as upper limb reaching [12]. Not only can these kinematic changes lead to performance errors during a given task (e.g. decreased precision while throwing a ball) [12], but they can also lead to an increased mechanical load on the periarticular structures, such as tendons, muscles and bursae [9, 10, 13]. It is thus important to better understand how and why such changes in shoulder movements occur.

Fatigue, defined as:”*a symptom in which physical and cognitive function is limited by interactions between performance fatigability and perceived fatigability”* [14, 15], is a daily encountered condition that could be responsible for the kinematic changes observed in individuals with chronic RCRSP. It has been demonstrated that during upper limb work related tasks such as hammering [16], ratcheting [17] and simulated chain work [18–20], fatigue leads to kinematic adaptations, including decreased glenohumeral elevation, and increased trunk and scapular movements [14, 15, 21]. These observed kinematics changes following fatigue could be caused by different modulating factors [14] such as the reduction of proprioception acuity [15], muscle activation latency [22] and/or muscle force variability [23, 24]. As kinematics changes could increase mechanical load on the shoulder joint and lead to injuries, it is important to better understand how fatigue influences shoulder movements, to assess its potential harmful effects and improve preventive interventions.

Overhead activities (above 60° of shoulder elevation) are frequently performed during daily life, work and sport activities. They are especially demanding for the shoulder because the stability of the glenohumeral joint decreases in elevated positions [1, 2]. The kinematic changes that are secondary to fatigue [14] could be even more detrimental for the shoulder periarticular structures during overhead activities. To our knowledge, existing studies have evaluated the impact of fatigue on shoulder kinematic during tasks performed at the waist level [20–24]. There is a need to better understand the impact of fatigue during tasks in elevated positions as it could help explain the development of shoulder pain.

The primary objective of this study was to explore how fatigue influences shoulder movements during an upper limb reaching task performed in an elevated arm position and in a virtual reality environment (VRE). Shoulder, elbow and trunk movements were characterized using kinematics (joint angles and excursions) and spatiotemporal (speed, initial angle of endpoint deviation [iANG], area under the curve [area] and final error [fERR]) data, while the presence of fatigue was evidenced using EMG activity. Although studies on this topic are sparse, it is reasonable to believe that fatigue will lead to an increase in trunk and sternoclavicular joint movements, and reductions in glenohumeral joint movement [18, 25–27] and movement speed and accuracy [18].

## Methods

### Participants

The study population consisted of healthy adults aged between 18 and 40 years with no movement restriction and no self-reported upper limb or neck pain and disability at the time of assessment. Potential participants were excluded if they had: 1) previous neck and upper limb surgery or fracture; 2) a history of glenohumeral dislocation (<12 months). Subjects were recruited through the institutional mailing list of *Université Laval* and through social media. The Sectorial Rehabilitation and Social Integration Research Ethics Committee of the CIUSSS-CN approved this study and all subjects provided informed written consent. The individual in this manuscript has given written informed consent (as outlined in PLOS consent form) to publish these case details.

### Study design

Participants took part in one laboratory session. They first completed a questionnaire on sociodemographics and the Edinburgh Handedness Inventory to establish hand dominance. They were randomly assigned to the Control group or the Fatigue group. Randomization was stratified by sex and used a block design (block size of 4) [32]. Thereafter, each participant was asked to complete the two phases of the experiment:

- Baseline phase: participants performed a unilateral reaching task in a VRE with their dominant arm.
- Post-experimental phase: participants performed the same unilateral reaching task in the VRE with their dominant arm. Participants in the Fatigue group completed the task following a validated experimental shoulder fatigue protocol [25] while participants in the Control group completed the task after a 10-minute break (similar to the average time it took to perform the fatigue protocol).

During the task, electromyographic, spatiotemporal and kinematic data of the upper limb were collected.

### Reaching task

**Fig 1:**
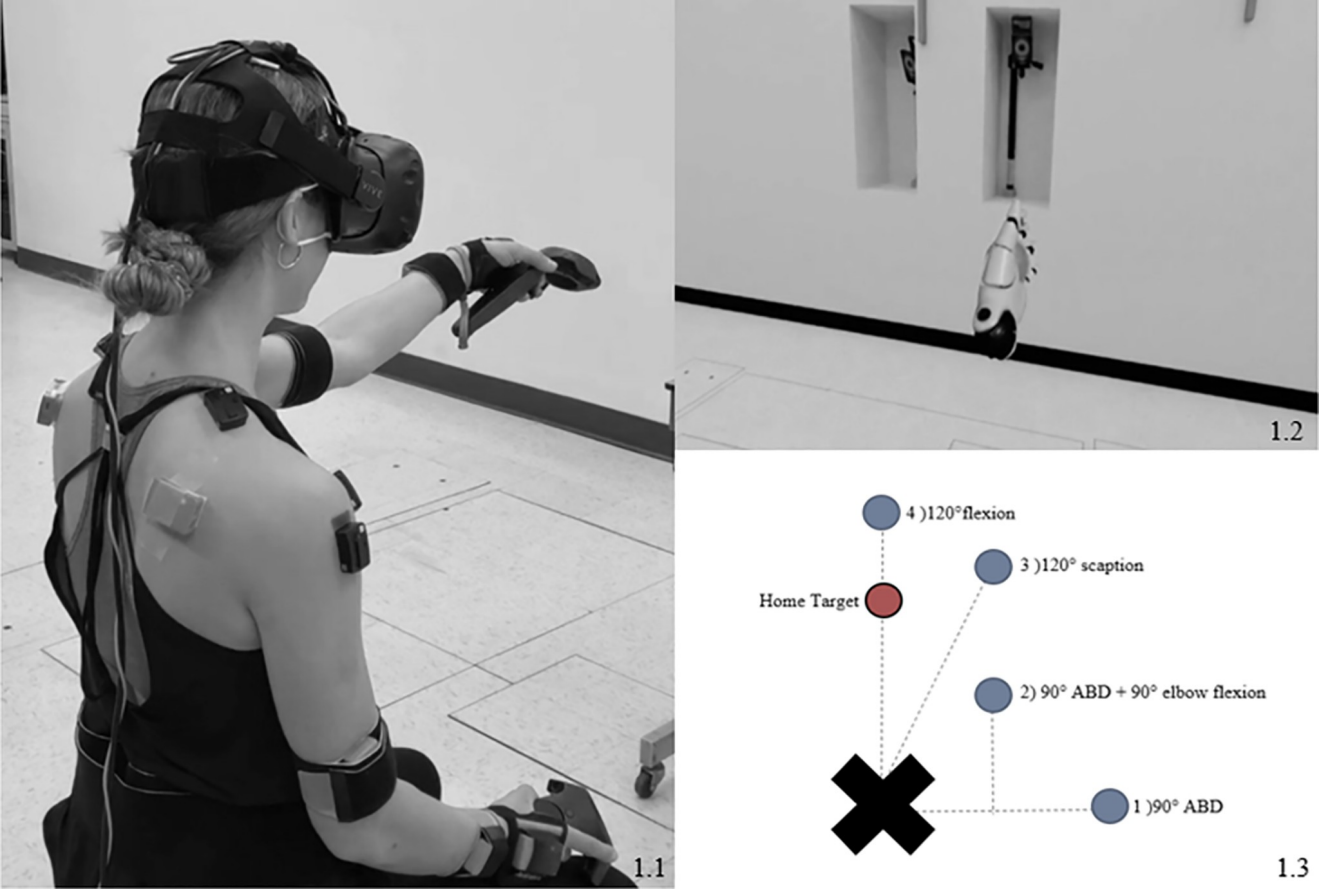
Targets position and set up. ABD: abduction. a: a participant performing the task with EMG sensors, IMUs and VivePro helmet and controllers; b: Participant view of its virtual hand while performing the task, left hand on home target; c: Top view of the positioning of the targets around the participant (right-handed). The Home target is positioned at 90° of shoulder flexion; Target 1 at 90° of shoulder abduction, elbow fully extended; Target 2 at 90° of shoulder abduction and 90° of shoulder external rotation, elbow flexed at 90°, Target at 120° of shoulder elevation in the scapular plan and Target 4 at 120° of shoulder flexion.

The task was conducted in a VRE, created with Unreal Engine (Epic games international, Unreal Engine, Switzerland). Participants were exposed to a realistic visual scene (the same room in which data were collected) with 3D depth information (Unreal 3) by wearing HTC VIVE goggles (HTC corporation, VIVEPORT, Taoyuan City, Taoyuan County, Taiwan) (Fig 1) [28]. They held a controller in their dominant hand that appeared to them as their real hand in the VRE and they could see a ball (2 cm radius) in the palm of their virtual hand. They completed the task seated on a chair, with the trunk free to move, knees flexed at 90° and feet on the floor. Participants were asked to move naturally without moving their feet during the task. Foot position was marked to ensure consistent foot placement throughout the trials.

The task consisted of a series of four virtual targets, visualized as balls with a 5-cm radius. The starting position was on the ‘home target’, placed at 90°of shoulder flexion, extended elbow, and neutral humeral rotation (Fig 1). The first virtual reaching target was then released in random order, and participants were instructed to reach that target as fast and accurately as possible, using the most direct path, and placing the virtual ball in the palm of their virtual hand on the middle of the target. The reaching target then disappeared, and the participants returned to the home target as fast as possible to release the next one. Each of the four reaching targets appeared five times and participants had to reach the total of the 20 targets without rest to complete the trial. The use of the VRE allowed the positioning of the targets in elevated positions, at different angles and in different movement planes, according to the participants’ anthropometrics (e.g. seated height and arm length). It also allowed a spontaneous and random appearance of the different targets.

Reaching target positions were set in the VRE at the following pre-established shoulder positions using an electronic inclinometer placed laterally to the humerus of the participants and a goniometer (Fig 1):

1. 90° humeral abduction (ABD), elbow extended.
2. 90° external rotation (ER) at 90° humeral of ABD, 90° flexed elbow.
3. 120° humeral elevation in the scapular plane, extended elbow, neutral humeral rotation.
4. 120° humeral elevation in the sagittal plane (pure flexion), extended elbow, neutral humeral rotation.

These different positions allowed us to assess the impact of fatigue during reaching in different elevated arm positions. Participants of both groups were asked to rate their perceived shoulder exertion level before and immediately after the reaching task using the Borg Rating of Perceived Exertion Scale (Borg CR10 Scale).

### Fatigue protocol

Participants in the Fatigue Group performed a previously validated experimental shoulder fatigue protocol [25] with their dominant arm. The protocol was composed of three activities: 1) manipulating screws on a wooden board for 2 minutes with the shoulders at 45° of flexion and with the elbows fully extended; 2) 20 repetitions of arm elevations in the sagittal plane holding a dumbbell (2 pounds for women; 4 pounds for men); and 3) 20 repetitions of arm elevations in the scapular plane holding the same a dumbbell. Borg scale perceived level of exertion was asked and recorded every 30 seconds during the fatigue protocol. Participants repeated these three activities until they reported reaching at least 8/10 on the Borg Rating of Perceived Exertion Scale. The post-experimental phase was performed immediately upon finishing the fatigue protocol.

### Instrumentation

Six inertial measurement units (IMUs) (MVN, Xsens Technologies, Enschede, Netherlands; sampling rate: 60 Hz) were used to measure joint angles during the task [29]. IMUs were placed on the head, sternum, pelvis, scapula, upper arm and forearm of the participant’s dominant arm. Sensors were placed in accordance with Xsens suggested sensors configuration using Velcro straps. Anthropometric measures (height, shoulder width, arm span, hip height, hip width, knee height, ankle height, foot size and sole height) were recorded and entered in the MVN Studio software (MVN studio software, v. 4.4.0, Xsens Technologies, Enschede, Netherlands). The Npose + walk calibration was used to calibrate the Xsens system as recommended by Xsens.

Wireless surface EMG sensors (Delsys Trigno, USA) were placed on the anterior and middle deltoids of the dominant arm. These two muscles were chosen because they are the main agonist in shoulder flexion and abduction. They are also highly implicated in shoulder movement above 90° elevation [30] and their EMG activities correlate with physical demands of working tasks [31]. The skin was cleaned using alcohol prior to electrode placement. The Surface EMG for Noninvasive Assessment of Muscles (SENIAM) guidelines were used to position the sensors [32]. The anterior deltoid sensor was placed 1–2 cm below the acromioclavicular joint and the middle deltoid sensor midway of deltoid insertion and acromion. Muscle activity was recorded using Delsys EMGworks^®^ Acquisition software (sampling rate: 1925.93Hz).

The controller held in the dominant hand and Unreal Engine were used to collect the spatiotemporal data during the task (sampling rate: 90 Hz) [28]. The three systems (Xsens, EMG and Unreal) were time-synchronized using a custom trigger box.

### Data analysis

For every trial, the mean values for kinematic and spatiotemporal variables were calculated for each target. The first eight reaching movements (1^st^ and 2^nd^ appearances of the 4 targets [8/20]) during the Baseline and Post-experimental phases were excluded from the analyses to decrease variability due to learning, as we did not perform a practice trial before the experimentation. Therefore, the mean of the last three reaching movements of each targets (12/20) was used for statistical analysis.

Joint angles were computed from the kinematic data using MVN studio and Xsens modified ISB body segment model. The variables of interest were 1) initial joint angles (joint angles on the home target between the reaching movements); 2) final joint angles (joint angles when the targets were reached) and 3) total joint angular excursions (final angle–initial angle). The analyzed joints were the trunk (lateral flexion [LF], rotation and flexion/extension), sternoclavicular joint (elevation), elbow (flexion-extension) and glenohumeral joint (flexion, ABD and ER). As the movement performed depended on target position, we used specific targets to evaluate specific joint movements: all targets (1, 2, 3 and 4) were used to assess sternoclavicular elevation, trunk flexion/extension and elbow flexion/extension as these movements were not influenced by the target position; targets number 1, 2 and 3 were used to assess trunk LF and rotation; targets 1 and 2 were used to assess the glenohumeral ABD; targets 3 and 4 were used to assess the glenohumeral flexion and target 2 was used to assess glenohumeral ER.

Fatigue was characterized as a downward shift in the EMG power spectrum (i.e. median power frequency [MDF]), associated with an increase in EMG signal amplitude, which has been proposed as good indicator of neuromuscular adaptation associated with fatigue [33]. All EMG signals were processed using custom software written in MATLAB R2013a (The MathWorks Inc., Natick, Massachusetts, United States). EMG signals were digitally filtered off-line with a zero-lag 4^th^ order Butterworth Filter (band-pass 20–450Hz). The band-passed signals were rectified, and their mean EMG signal amplitude (RMS, 10 ms non-overlapping window) and MDF were calculated in 20 seconds cycles for the two muscles evaluated. The mean values of the 20 seconds cycles were use for statistical analysis, as it allowed to assess the presence of EMG sign of fatigue across trials in a given phase. The power spectrum density was computed from the squared Fast-Fourier Transform [34].

**Fig 2:**
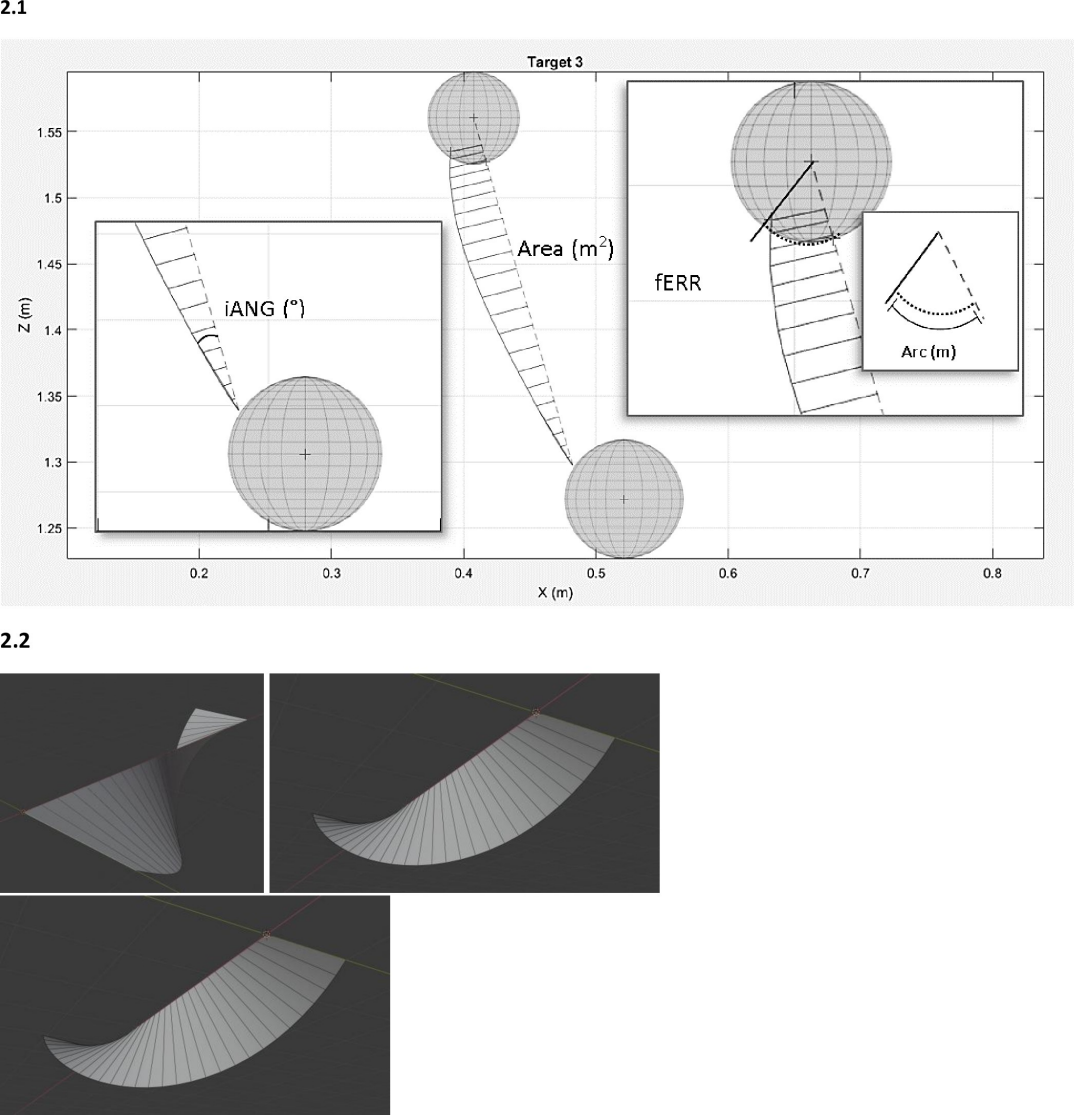
Spatiotemporal data collection. a. Example of spatiotemporal data collection while reaching target 3. iANG: initial angle of endpoint deviation, this angle was calculated using the shortest line between two targets [home and reaching targets] and the line corresponding to the initial peak of acceleration); fERR: final error, the shortest arc distance between the ideal arrival point into the sphere (target) and the actual arrival point. b. Example of a calculated 3D area under the curve for 1 reaching movement: the area under the curve is the summation of the rectangular trapezoids perpendicular to the ideal trajectory line and the actual trajectory line.

**Fig 3:**
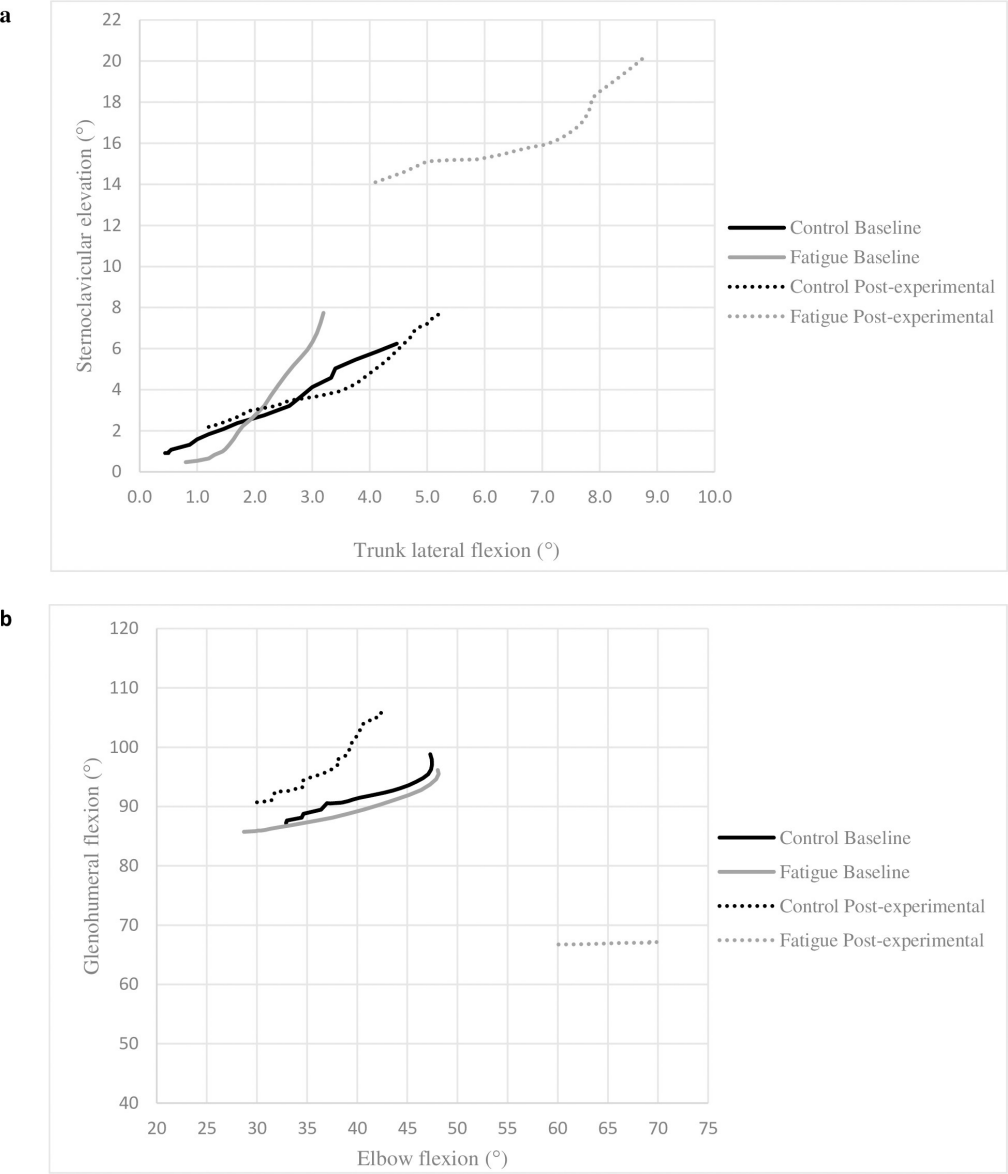
Inter-joint coordination at baseline and post-experimental phase. Inter-joint coordination while reaching target 2 (120° scaption). a. Trunk lateral flexion (LF) vs sternoclavicular (SC) elevation and b. Glenohumeral (GH) flexion vs elbow flexion. Trunk lateral flexion: positive value represents a LF ipsilateral to the reaching arm. Significant difference (Time x Group effect: p < 0.05) for SC elevation, trunk LF, GH flexion and elbow flexion between the phases. a. Fatigue group showed a significant increase of SC elevation and trunk LF initial and final positions and total excursion during post-experimental phase (p < 0.05). b. Fatigue group showed a significant decrease of GH flexion initial and final positions and an increase of elbow flexion initial and final positions; total joint excursion during post-experimental phase was decreased for both joints (p < 0.05).

**Fig 4:**
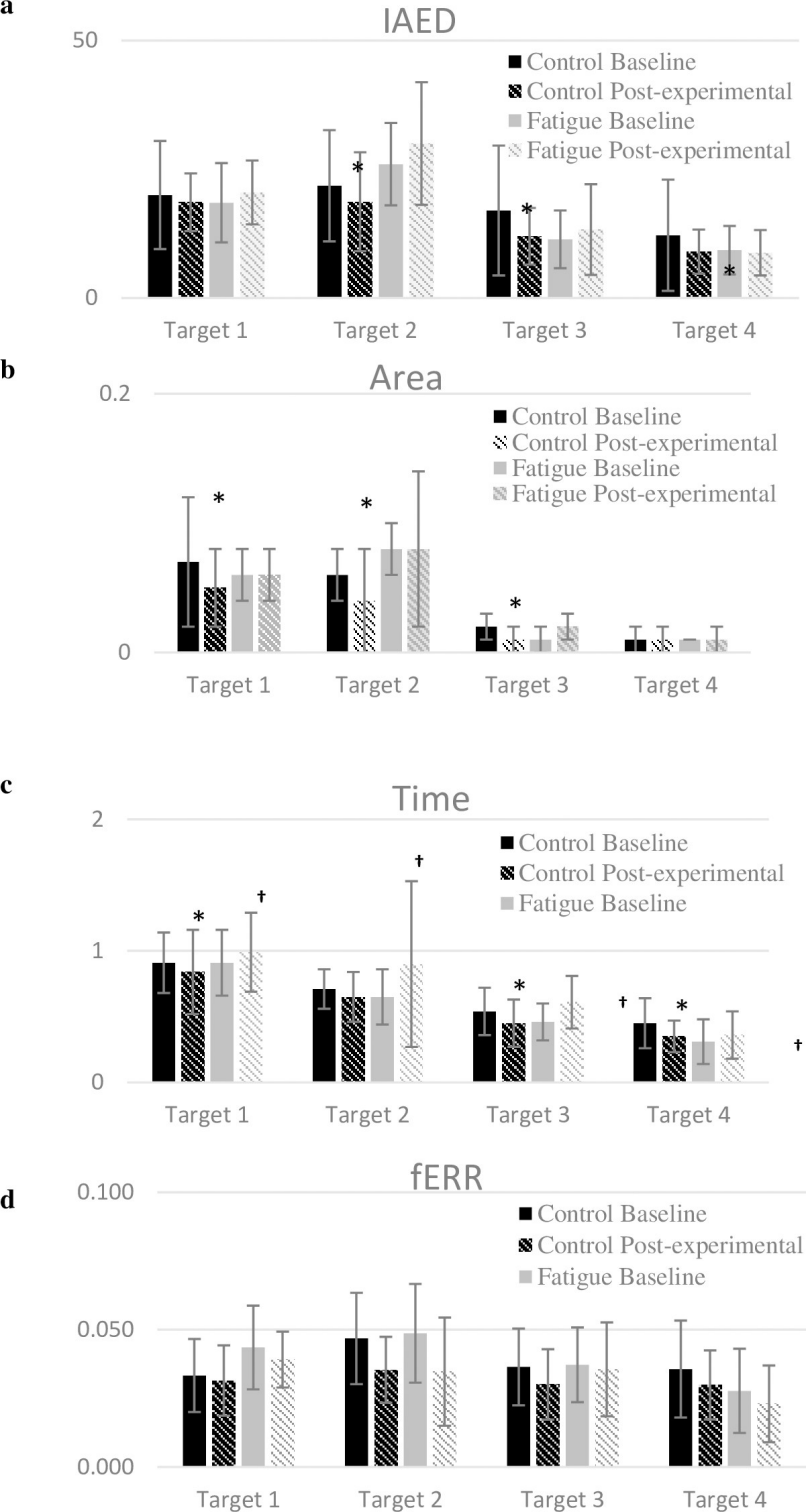
Spatiotemporal data during baseline and post-experimental condition of both groups. Data are presented as mean±SD of Baseline and Experimental condition for both groups. a: iANG, initial angle of endpoint deviation (°); b: Area, area under the curve (m^2^); c: Time, time taken to reach the targets (s); d: fERR, final error, arc sign (m). Significant Time effect and Time x Group effect, p<0.05, for iANG, Time and Area. No significant difference for fERR (Time effect: p = 0.226; and Time x Group effect: p = 0,33). *Significant increase of performance for controls compared to baseline, p < 0.05. ^†^Significant decrease of performance compared to baseline, p < 0.05.

Spatiotemporal outcomes were used to characterize the performance of the participants during the reaching task. They were obtained using Unreal Engine and extracted using a custom software written in MATLAB. There were 4 spatiotemporal variables of interest (Fig 2): 1) The time taken to reach the targets which is the time from the moment the target was released from the ‘‘home target” to reaching the reaching target; 2) The initial angle of endpoint deviation (iANG) which reflects movement planning as it was based on the initial trajectory of the hand; this angle was calculated using the shortest line between two targets (home and reaching targets) and the line corresponding to the initial peak of acceleration (Fig 2); 3) The final error (fERR) which reflected the accuracy of the movement; the shortest arc distance between the ideal arrival point into the sphere (target) and the actual arrival point (Fig 2); 4) The area under the curve representing total movement error which is the summation of the rectangular trapezoids perpendicular to the ideal trajectory line and the actual trajectory line (Fig 2).

### Statistical analyses

Baseline demographic data were compared between groups using independent t-tests and χ2. Perceived levels of fatigue on Borg rating scale were compared using a two-way repeated measures ANOVA to calculate the effect of Time (Baseline, Post-experimental) and Group (Fatigue, Control), as well as the interaction between these factors (proc GLM, SPSS 26). For EMG variables (signal amplitude and MDF), three-way repeated-measures ANOVA was used to calculate the effect of Time (Baseline, Post-experimental), Group (Fatigue, Control) and Cycle (20s cycles/trial) and the interaction effect between these factors. For spatiotemporal data and joint kinematics, a three-way repeated-measures ANOVA was used to calculate the effect of Time (Baseline, Post-experimental), Group (Fatigue, Control) and Target (Target 1, 2, 3 and 4), as well as the interaction effect between Time and Group. Inherent post-hoc tests were conducted to detail interactions between factors. All statistical tests were conducted in IBM SPSS Statistics (IBM SPSS Statistics 26, IBM Corp., NY, USA) with a significance level set at 0.05.

## Results

Forty healthy participants were included: 20 in each group. Table 1 presents their baseline characteristics; there were no significant differences between the groups (p > 0.05).

**Table 1:** Participant’s baseline characteristics.

	Control group (n = 20)	Fatigue group (n = 20)
**Age, years,** $\bar{X}$**±SD**	26±3	26±3
**Sex, male (%)**	9 (45)	7 (35)
**Height, cm,** $\bar{X}$**±SD**	170.4±9.4	172.5±9.3
**Weight, kg,** $\bar{X}$**±SD**	66.1±11.8	72.9±11.4
**Dominant side, right (%)**	20 (100)	18 (90)
**Sports**^**†**^**, n (%)**	10 (50)	12 (60)
**Work**^**†**^**, n (%)**	9 (45)	12 (60)

$\bar{X}$, mean.

^†^: Participants were asked if they were practicing a sport or/and having a job with a physical requirement of the shoulder (yes/no)

No significant difference between the groups for all characteristics (independent t-test and χ2, p > 0.05)

### Fatigue

#### Perceived fatigue

Participants in both groups started the Baseline phase with a mean perceived level of fatigue of 0 and ended this phase with a mean score of 3±1on the Borg scale. Participants in the Fatigue group completed the fatigue protocol in 6.5±2.0 minutes with a mean fatigue score of 9.0±0.6. When participant reached 6-7/10, the perception of fatigue became exponential, which explains that most participants quickly went from a 6-7/10 to 9/10.

Participants in the Fatigue group started the Post-experimental phase with a significant higher mean perceived level of fatigue, ending with a higher mean score compared to baseline, while participants in the Control group showed no difference in mean values between the phases (Time x Group effect: p < 0.001; Fatigue group: mean score before the Post-experimental phase = 7±3 and after the Post-experimental phase = 8±2; Control group: mean score before the Post-experimental phase = 0 and after the Post-experimental phase = 3±1).

#### EMG activity

EMG analysis showed that anterior deltoid was fatigued in the Post-experimental phase in the Fatigue Group. There was a significant Time x Group interaction (p < 0.012) for the EMG amplitude and MDF. In the Fatigue group, mean median power frequency of the anterior deltoid was significantly decreased, while the mean EMG amplitude was significantly increased (p < 0.01); in contrast, the Control group did not show significant changes for these EMG variables between the phases (p > 0.05, Table 2).

**Table 2:** EMG signals of the two groups during baseline and experimental phase.

	Anterior Deltoid		Middle Deltoid	
	Amplitude (V)·10^−5^	MDF (Hz)	Amplitude (V)·10^−5^	MDF (Hz)
**Control Baseline**				
0-20s	5.8 (2.1)	95.5 (23.2)	5.9 (3.7)	71.4 (9.4)
20-40s	6.1 (2.2)	95.8 (23.1)	6.4 (4.1)	71,8 (9.4)
40-60s	6.5 (2.3)	94.9 (23.1)	6.5 (3.9)	71.3 (8.4)
**Fatigue Baseline**				
0-20s	6.6 (3.2)	89.6 (22.1)	4.7 (3.2)	69.9 (14.4)
20-40s	7.2 (3.2)	87.5 (21.6)	5.2 (3.5)	70.8 (14.9)
40-60s	7.1 (3.2)	85.6 (20.9)	5.3 (3.7)	70.7 (14.1)
**Control Post-exp**				
0-20s	6.0 (3.7)*	98.4 (23.7)*	5.7 (3.7)*	74.1 (9.9)
20-40s	6.6 (2.4)*	96.7 (22.8)*	6.0 (3.7)*	73.4 (9.8)^**†**^
40-60s	6.7 (2.6)*	96.6 (24.0)*	6.4 (3.9)*	72.6 (9.8)^**†**^
**Fatigue Post-exp**				
0-20s	8.1 (3.7)*^†^	81.9 (20.1)*^†^	6.5 (5.0)*^†^	70.3 (11.8)
20-40s	8.8 (4.3)*^†^	82.7 (20.2)*^†^	6.7 (4.8)*^†^	72.5 (13.7)^**†**^
40-60s	9.8 (2.6)*^†^	82.7 (21.0)*^†^	7.4 (5.4)*^†^	73.1 (15.2)^**†**^

• Post-exp: post-experimental phase; MDF: mean median power frequency.

• Data are presented as mean (SD).

• ^†^Significant difference between baseline and post-experimental phase, p < 0.05.

• *Significant Time x Group interaction, p < 0.05.

The mean EMG amplitude of the middle deltoid increased significantly in the Fatigue group between baseline phase and post-experimental phase (Time x Group interaction, p = 0.08), while it remained stable in the Control group (p > 0.09; Table 2). There was also a significant Cycle effect for both groups (p = 0.001) as the middle deltoid EMG amplitude increased significantly during the trials (p = 0.001). There was however no significant Time x Group interaction for the mean median power frequency of the middle deltoid (p = 0.768). A Time effect (p = 0.009) was observed for the MDF of the middle deltoid as it increased in both groups (p < 0.01; Table 2).

### Joint kinematics

Significant Time x Group interactions (p < 0.01) were observed for all initial joint positions (in the home target) (except for glenohumeral ABD; p = 0.97), as well as for total joint excursions and final joint positions (joints amplitude when the targets were reached). Post hoc tests showed that participants in the Fatigue group made significant modifications in their global posture (initial joint angles) as well as in their reaching movement strategy (final joints angles and total joints angular excursions) in the fatigued state, while the Control group did not show significant changes between phases.

Changes in initial joint angles in the fatigued state included increased trunk flexion (p < 0.04), rotation (p < 0.01) and LF ipsilateral to the reaching arm (p = 0.01), as well as increase elbow flexion (p < 0.001) and glenohumeral ER (p < 0.001). Glenohumeral flexion was decreased (p < 0.001), which may have been compensated by the increase sternoclavicular elevation noted (p < 0.04, Table 3).

**Table 3:** Mean total joint excursions during baseline and experimental phase of both groups.

	Target	Initial joint angles Mean (SD)	*p*	Final joint angles Mean (SD)	*p*	Total angular excursion Mean (SD)	*p*
**Trunk LF**			0.001*		0.05*		0.002*
Control Baseline	1	2.3 (2.1)		7.8 (2.6)		4.4 (4.6)	
	2	2.2 (2.3)		5.5 (2.5)		2.9 (3.6)	
	3	2.3 (2.1)		4.1 (1.5)		1.5 (2.0)	
Control post-exp	1	2.1 (2.1)		8.0 (2.8)		4.8 (4.3)	
	2	2.1 (2.2)		5.4 (2.6)		2.9 (3.5)	
	3	2.0 (2.4)		4.1 (2.1)		1.8 (2.0)	
Fatigue Baseline	1	2.5 (2.1)		9.1 (2.3)		6.5 (1.7)	
	2	2.5 (1.9)		6.3 (1.9)		3.8 (2.0)	
	3	2.6 (1.9)		4.6 (2.1)		2.0 (1.5)	
Fatigue post-exp	1	4.5 (2.4) ^**†**^		10.2 (2.2)		5.7 (2.1) ^**†**^	
	2	4.6 (2.5) ^**†**^		7.1 (2.1)		2.5 (2.1) ^**†**^	
	3	4.7 (2.2) ^**†**^		6.1 (1.9) ^**†**^		1.4 (1.2) ^**†**^	
**Trunk rotation**			0.005*		0.005*		0.758
Control Baseline	1	-13.7 (4.6)		-13.7 (4.6)		-10.8 (6.1)	
	2	-13.0 (5.6)		-13.0 (5.6)		-10.4 (5.8)	
	3	-7.9 (5.7)		-7.9 (5.7)		-5.4 (4.2)	
Control post-exp	1	-13.9 (7.5)		-13.9 (7.5)		-11.9 (8.1)	
	2	-12.8 (5.9)		-12.8 (5.9)		-10.9 (6.2)	
	3	-6.0 (5.5)		-6.0 (5.4) ^**†**^		-4.7 (3.6)	
Fatigue Baseline	1	-15.0 (5.8)		-15.0 (3.8)		-12.1 (2.0)	
	2	-15.7 (3.6)		-14.7 (3.6)		-11.6 (1.8)	
	3	-8.3 (4.4)		-8.3 (4.4)		-5.3 (3.0)	
Fatigue post-exp	1	-16.6 (2.8)^**†**^		-16.6 (2.8) ^**†**^		-11.5 (1.6)	
	2	-16.7 (2.8) ^**†**^		-16.7 (2.8) ^**†**^		-11.3 (1.8)	
	3	-11.4 (4.9) ^**†**^		-11.4 (3.4) ^**†**^		-6.3 (1.6)	
**Trunk flexion**			0.001*		0.028*		0.044*
Control Baseline	1	18.5 (4.3)		17.3 (3.7)		-0.7 (2.3)	
	2	18.7 (3.5)		12.5 (3.1)		-5.3 (4.2)	
	3	18.5 (4.3)		14.3 (3.0)		-3.4 (2.5)	
	4	18.4 (4.1)		15.5 (3.4)		-2.5 (2.4)	
Control post-exp	1	17.4 (3.9)		16.9 (3.6)		0.1 (2.2) ^**†**^	
	2	17.4 (4.0)		11.6 (3.6) ^**†**^		-4.9 (3.9)	
	3	17.3 (4.2) ^**†**^		14.2 (3.6)		-2.7 (2.2)	
	4	17.4 (4.1) ^**†**^		14.8 (3.9)		-2.2 (2.3)	
Fatigue Baseline	1	18.9 (2.4)		18.0 (2.3)		-0.8 (2.1)	
	2	18.6 (4.4)		13.3 (2.5)		-5.7 (2.2)	
	3	18.9 (2.2)		15.6 (2.3)		-3.3 (1.1)	
	4	18.6 (2.4)		16.3 (2.8)		-2.4 (1.6)	
Fatigue post-exp	1	20.3 (3.6) ^**†**^		18.6 (3.5)		-1.7 (1.8) ^**†**^	
	2	20.4 (3.4) ^**†**^		13.5 (3.5)		-6.9 (2.1) ^**†**^	
	3	20.4 (3.5) ^**†**^		16.6 (3.8)		-3.9 (2.9)	
	4	19.8 (3.6)		18.2 (3.9) ^**†**^		-1.6 (2.1)	
**Elbow flexion**			0.006*		0.082		0.048*
Control Baseline	1	25.1 (15.6)		17.l0 (12.9)		-7.3 (9.6)	
	2	23.9 (1.3)		78.0 (13.5)		54.1 (12.0)	
	3	24.1 (16.2)		25.5 (15.6)		1.6 (6.8)	
	4	24.3 (15.9)		32.4 (17.6)		6.7 (8.4)	
Control post-exp	1	27.9 (16.7)		18.2 (12.7)		-8.6 (14.0)	
	2	26.9 (16.2)		80.3 (13.1)		53.4 (14.5)	
	3	27.2 (16.6)		29.1 (18.4)		1.0 (12.8)	
	4	27.0 (17.2)		34.6 (19.7)		6.6 (12.1)	
Fatigue Baseline	1	26.8 (13.4)		16.4 (14.4)		-10.4 (9.7)	
	2	26.0 (13.8)		78.8 (12.9)		52.8 (13.7)	
	3	27.1 (14.2)		26.7 (15.6)		-0.3 (8.1)	
	4	26.3 (13.6)		34.0 (14.1)		7.7 (7.8)	
Fatigue post-exp	1	42.1 (18.3) ^**†**^		23.9 (14.1) ^**†**^		-18.2(13.7) ^**†**^	
	2	41.1 (18.3) ^**†**^		82.6 (11.1) ^**†**^		41.5 (17.3) ^**†**^	
	3	41.6 (19.1) ^**†**^		36.4 (14.6) ^**†**^		-5.2 (14.3)	
	4	42.7 (18.1) ^**†**^		45.9 (14.6) ^**†**^		3.2 (10.9)	
**GH ABD**			0.967		0.019*		0.003*
Control Baseline	1	33.1 (9.7)		71.8 (8.0)		34.7 (19.0)	
	2	33.0 (9.7)		68.0 (10.5)		32.1 (16.5)	
Control post-exp	1	32.1 (10.9)		71.0 (9.1)		35.3 (19.4)	
	3	31.3 (11.1)		66.5 (12.8)		32.9 (16.0)	
Fatigue Baseline	1	32.4 (10.6)		72.1 (8.4)		39.8 (7.1)	
	2	30.9 (10.7)		61.7 (10.9)		30.8 (6.7)	
Fatigue post-exp	1	30.3 (10.8)		60.9 (10.7) ^**†**^		30.6 (7.1) ^**†**^	
	2	30.6 (10.9)		60.0 (11.8)		29.3 (7.9)	
**GH flexion**			**<**0.0001*		<0.0001*		0.028*
Control Baseline	3	75.9 (11.5)		96.9 (16.1)		21.0 (8,4)	
	4	76.0 (11.6)		95.6 (11.9)		19.6 (5.3)	
Control post-exp	3	74.9 (13.2)		95.1 (19.0)		20.2 (8.7)	
	4	75.2 (13.4)		94.8 (13.2)		19.6 (6.4	
Fatigue Baseline	3	75.4 (9.5)		99.8 (11.6)		22.8 (9.1)	
	4	76.7 (8.3)		99.1 (9.2)		21.9 (6.1)	
Fatigue post-exp	3	53.9 (11.1) ^**†**^		73.1 (13.5) ^**†**^		17.9 (9.3) ^**†**^	
	4	54.3 (10.8) ^**†**^		76.4 (11.3) ^**†**^		21.6 (10.4)	
**GH ER**			<0.0001*		0.165		0.093
Control Baseline	1	20.5 (10.0)		-13.7 (32.9)		-30.4 (34.2)	
Control post-exp	1	20.6 (10.6)		-17.4 (37.6)		-34.8 (38.7)	
Fatigue Baseline	1	19.5 (14.1)		-4.6 (20.3)		-24.1 (21.4)	
Fatigue post-exp	1	7.2 (14.7) ^**†**^		-19.1 (17.6)		-26.3 (13.3)	
**SC elevation**			<0.0001*		<0.0001*		0.009*
Control Baseline	1	-5.5 (4.1)		2.7 (4.2)		7.1 (5.6)	
	2	-5.6 (4.1)		7.3 (4.7)		11.2 (7.8)	
	3	-5.6 (4.1)		2.0 (4.5)		6.1 (5.5)	
	4	-5.8 (3.9)		-0.3 (4.3)		4.7 (3.6)	
Control post-exp	1	-5.4 (4.5)		1.8 (4.7)		6.0 (5.4)	
	2	-5.3 (4.7)		6.1 (5.1)		9.6 (7.6)	
	3	-5.3 (4.6)		0.86 (4.2)		5.3 (3.9)	
	4	-5.6 (4.8)		-0.5 (4.9) ^**†**^		4.6 (2.8)	
Fatigue Baseline	1	-5.3 (3.8)		1.3 (4.1)		6.6 (6.4)	
	2	-4.6 (4.8)		5.1 (5.0)		9.7 (7.9)	
	3	-5.4 (3.8)		0.9 (4.7)		6.3 (4.7)	
	4	-5.6 (3.5)		-0.5 (5.0)		5.1 (4.2)	
Fatigue post-exp	1	-0.5 (3.8) ^**†**^		9.1 (4.6) ^**†**^		9.6 (2.9) ^**†**^	
	2	-2.9 (4.8) ^**†**^		11.3 (4.5) ^**†**^		12.5 (2.4) ^**†**^	
	3	-0.7 (4.1) ^**†**^		7.6 (4.1) ^**†**^		8.3 (2.0)	
	4	-0.9 (3.8) ^**†**^		5.6 (5.3) ^**†**^		6.4 (4.1)	

• LF: Lateral Flexion; SC: sternoclavicular; GH: glenohumeral; ER; external rotation; ABD; abduction; post-exp: post-experimenta, fatigued state for the fatigue group.

• Trunk rotation: Negative value represents a trunk rotation ipsilateral to the reaching arm; LF: Positive value represents a LF ipsilateral to the reaching arm; ER: negative values represent external rotation, positive values represent internal rotation.

• Target 1: 90° glenohumeral ABD, elbow extended; Target 2: 90° glenohumeral ABD and 90° glenohumeral ER; Target 3: 120° scaption; Target 4: 120° glenohumeral flexion

• Data are presented as mean value (SD)

• *significant Time x Group interaction (p < 0.05).

^†^A statistically significant change in mean score compared with baseline values (p < 0.05)

In the fatigued state, the participants in the Fatigue Group used more trunk and sternoclavicular movements and less glenohumeral movements to reach the targets, as evidenced by increased trunk extension (p < 0.03), LF (p < 0.01) and sternoclavicular elevation (p < 0.04) total angular excursion and a decrease of glenohumeral ABD (p < 0.001) and glenohumeral flexion (p < 0.05) total angular excursion (Table 3). It resulted in an increase of their final sternoclavicular elevation (p < 0.001) and trunk ipsilateral LF (p < 0.01) angles (Fig 3A) and a decreased of their final glenohumeral ABD and glenohumeral flexion angles (p < 0.001, Fig 3B). The Fatigue Group also maintained more elbow flexion while reaching to the targets, resulting in a decreased total elbow angular excursion during the Post-experimental phase (Fig 3B).

### Spatiotemporal

There was a significant Time x Group interaction for all spatiotemporal data (iANG, the areas under the curve and the time) (p < 0.04, Table 4), but fERR (p = 0.33). The Control Group improved their task performance, as they decreased movement errors (iANG and area under the curve) and time taken to reach the targets during the Post-experimental Phase (p < 0.05, Fig 4). Such improvement was not observed in the Fatigue Group as their performance remained stable regarding movement error (iANG) and area under the curve (p > 0.41, Fig 4). The time taken to reach the targets was longer in the Fatigue Group during post-experimental phase compared to baseline (Time effect: p = 0.03). The final error, however, did not change in both groups in the Post-experimental phase when compared to their Baseline phase (Time effect, p = 0.226).

**Table 4:** Spatiotemporal data mean difference between baseline and experimental condition of both groups.

Spatiotemporal data	Target 1	Target 2	Target 3	Target 4
**iANG Mean difference**				
Control	-1.38(1.70)	-3.15(1.76) *	-5.03(2.34) *	-3.24(2.32) *
Fatigue	1.97(1.29)	3.94(3.02)	1.83(1.85)	-0.44(1.08)
**Area Mean difference**				
Control	-0.019 (0.010) *	-0.010 (0.005) *	-0.005 (0.003) *	-0.002 (0.001)
Fatigue	0.002 (0.006)	0.007 (0.011)	0.002 (0.003)	0.001 (0.001)
**Time Mean difference**				
Control	-0.069 (0.050) *	-0.067 (0.049)	-0.086 (0.032) *	-0.11 (0.031) *
Fatigue	0.076 (0.073) ^**†**^	0.25 (0.130) ^**†**^	0.15 (0.056) ^**†**^	0.064 (0.047) ^**†**^
**fERR Mean difference**				
Control	-0.01 (0.01)	-0.01 (0.01)	-0.01 (0.01)	-0.01 (0.01)
Fatigue	-0.05 (0.19)	-0.10 (0.40)	-0.04 (0.16)	-0.05 (0.18)

• iANG: initial angle of endpoint deviation (°); Area: area under the curve (m^2^); Time: time taken to reach the targets (s); fERR: final error, arc sign (m). Target 1: 90° glenohumeral ABD, elbow extended; Target 2: 90° glenohumeral ABD and 90° glenohumeral ER; Target 3: 120° scaption; Target 4: 120° glenohumeral flexion.

• Mean difference: mean difference between baseline and experimental phase. Data are presented as mean difference (SEM).

• Significant Time effect and Time x Group effect, p<0.05, for iANG, Time and Area under the curve. No significant difference for fERR (Time effect: p = 0.226; and Time x Group effect: p = 0,33)

• *Significant increase of performance for controls compared to baseline, p < 0.05.

• ^†^Significant decrease of performance compared to baseline, p < 0.05.

## Discussion

This study evaluated the impact of fatigue on shoulder movements during an overhead reaching task, representative of the type of movement frequently performed during daily life, work and sport activities. The use of a VRE allowed the evaluation of reaching movements toward targets positioned at 90° of shoulder elevation or above and in different planes of movement which, to our knowledge, has not been evaluated previously. Following a fatigue protocol mostly performed in the sagittal plane, fatigue was induced in the anterior, but not the middle deltoid. This observation was associated with changes in both kinematic and spatiotemporal variables.

### Fatigue and joint kinematics

Upper limb kinematics were influenced by fatigue as there were significant changes in movement patterns including a different initial posture and a different reaching strategy. The changes in the fatigued state included decreased glenohumeral elevation, which seems to have been compensated by increased excursion at the trunk and sternoclavicular joint. Other studies obtained similar kinematic changes while performing working tasks [18–20], ratcheting [17] and reaching movements [35]. It was suggested that these kinematic changes aimed to reduce the load on the fatigued joint (i.e. glenohumeral muscles) [28]. Our results are consistent with this hypothesis, as from a biomechanical point of view, both postural adaptations (i.e. trunk and sternoclavicular joints) and arm kinematics (i.e. glenohumeral and elbow joints) changes appear to decrease the load on the glenohumeral joint.

Shoulder elevators, such as the deltoid, have been shown to be affected by fatigue during arm elevation tasks [17, 30], such as the fatigue protocol used in the present study. In fact, the fatigue protocol led to the desired effect as fatigue was evident in the anterior deltoid. The decrease in glenohumeral elevation observed in the fatigued state may be aimed at reducing muscular demands on the fatigued glenohumeral elevators [30]; similarly, the increase in elbow flexion and trunk ipsilateral rotation range of motion allowed to perform the reaching task while decreasing the lever arm of the glenohumeral elevators (i.e. flexor and abductor). These protective changes could have been compensated by increasing sternoclavicular elevation and trunk extension in order to reach the target [18].

The middle deltoid, the principal abductor muscle of the glenohumeral joint, did not show a significant decrease of its mean power frequency (although the mean amplitude was increased) [36]. As the home target was located in front of the participants (90° of humeral flexion), the principal elevation component of the whole task was in the sagittal plan. The fatigue protocol also included two of the three tasks in a sagittal plan, which can explain the lack of fatigue in the middle deltoid as opposed to the anterior deltoid. It may also explain the absence of significant changes in regard to glenohumeral ABD total excursion.

The reaching strategy (total joint angular excursions) also significantly differed in the fatigued state. Previous findings showed an inter-joint coordination reorganization of the upper limb similar to a rigid system after fatigue; it has been shown that in a fatigued state, there is less total angular excursion of the arm (i.e. wrist, elbow, shoulder) to complete upper limb tasks [17, 26, 35]. It led to the hypothesis that joint kinematic adaptations in a fatigued state are the result of a learning process in response to a new body state: in order to complete the movement in a new situation (i.e. new mechanical constraints), a new inter-joint coordination with fewer degrees of freedom occurs to decrease movement complexity [17, 37]. Our results are consistent with this hypothesis as there was less glenohumeral and elbow total angular excursion in the fatigued state. It suggests that the CNS adopted a simplified inter-joint coordination pattern to complete the task.

### Fatigue and spatiotemporal data

The Fatigue Group did not show improvement in task performance (as evidenced by the spatiotemporal variables) while the Control Group did (iANG, time and Area under the curve). Most of the current evidence on shoulder movement performance in a fatigued state suggests that task performance can be maintained by adopting new kinematic strategies, such as new inter-joint coordination [35, 37–40]. Our results are consistent with previous findings which showed that final hand positions remained accurate during an upper limb reaching task in a fatigued state [38, 40]: in the present study, the final error was not significantly different in the fatigued state compared to baseline and to the Control Group. These results could also be explained by the fact that the fatigue protocol mainly focused on the anterior deltoid, while the task included three of the four targets in the frontal/scaption planes. Thus, movement accuracy may have remained stable because the muscles recruited during the task (e.g., the middle deltoid) were not those most affected by the fatigue protocol. However, even if movement accuracy (iANG, fERR, Area) remained stable during post-experimental phase, the fatigue group decreased their movement speed likely to accurately complete the task.

As the Control Group improved their performance, but not the Fatigue Group [37, 41], the lack of performance improvement for the latter may be explained by the fact that fatigue introduced new mechanical constraints on the body that the CNS had to integrate to adapt movement planning: this new body state places the CNS in a new learning process that could interfere with performance. For example, the changes in posture following fatigue could be seen as a new body state that may have altered the performance metrics [37, 41].

Proprioception has been shown to be altered in the presence of fatigue [42, 43], which suggests that visual feedback becomes even more important in the fatigued state to perform a reaching task. Thus, to maintain the movement accuracy, visual feedback may be used to a greater extent to guide the motor commands in the fatigued-state [39], resulting in an increase of the mean time to accurately reach the targets (feedback control). It would have been interesting to include a group without visual feedback to evaluate these mechanisms, as a previous study showed that the lack of visual feedback had more impact on movement accuracy than fatigue during a reaching task [41]. Fatigue seems to impact performance by challenging the CNS with the resulting mechanical constraints, but more studies are needed to better understand the mechanisms as there could be muscular, spinal or/and central effects.

### Possible consequence of kinematic adaptations and clinical perspectives

The consequences of the adaptations in a fatigued state on shoulder structures (i.e., tendons, bursa, muscles) are unknown, but they are similar to those evidenced in glenohumeral and scapular kinematics in individuals suffering from chronic RCRP, which have been proposed to cause a reduction of the acromiohumeral distance and the compression of subacromial structures (bursa, rotator cuff and long heap of the biceps tendons) and to increase mechanical load on these structures [9, 11, 13]. In fact, in healthy individuals, fatigue has been shown to lead to an immediate reduction of the acromiohumeral distance [44–46]. As previously mentioned, the reason why these changes occur in injured populations is not clear, so the question is: can adaptations observed in a fatigued state, if repeated, be responsible for long-term modifications in shoulder kinematics. As we demonstrated that fatigue affects shoulder movement and performance, we can hypothesize that movement repetitions in a fatigued state might lead to a consolidation of maladaptive motor patterns, leading to higher physical stress on tendons and muscles. This is consistent with the findings that working above shoulder level (i.e. >90° of shoulder elevation or hand above shoulder level) without physical pauses (i.e. lack of micro pause in shoulder flexion [≤80% and >80% of the cycle time]) is a risk factor for development of shoulder RCRP [47]. It would be interesting to investigate the effect of a specific shoulder elevator endurance training program on adaptation to fatigue, as it has the potential to prevent the observed kinematic compensations in a fatigued state and thereby, reduce the risk of shoulder injuries.

### Strengths and limitations

This study has several strengths including having a control group that allowed considering the effect of repeatedly performing the task on the variables of interest. It also evaluated task performance using novel parameters (fERR, iANG, Area, time) allowing a more in-depth analysis of the impact of fatigue on movement’s spatiotemporal characteristics. Finally, to our knowledge, no study had yet explored the impact of fatigue during an upper limb task in elevated position, a demanding position for shoulder muscular control [2].

There are also some limitations to our study. First, all participants were young (between 18–40 years old) and free from shoulder pain. Therefore, generalization to older populations or to populations with shoulder pain is limited. Secondly, participants performed the task in a virtual reality environment, which could affect sensory perception. Finally, the adaptations induced by experimental fatigue may not be identical to the actual adaptations occurring in a real-life situation.

## Conclusion

This study aimed at investigating the impact of fatigue on shoulder movements during an upper limb reaching task in elevated arm positions. Participants in the Fatigue group showed postural adjustments and a new movement pattern to complete the task including less glenohumeral movement and more trunk and sternoclavicular movements. Performance was altered as shown by the lack of accuracy improvement over time and a decrease in movement speed in the Fatigue group. Based on these results, fatigue had physiological impacts which led to new mechanical constraints challenging the CNS, and as observed when learning a new movement, there was a new simplified inter-joint coordination pattern. This study therefore highlights the importance of considering fatigue as a potential factor influencing shoulder movements. Future work is now needed to understand how fatigue impacts movement control at muscular, spinal, and central levels.
